# Assessment of Current Mental Health Status in a Population-Based Sample of Canadian Men With and Without a History of Prostate Cancer Diagnosis: An Analysis of the Canadian Longitudinal Study on Aging (CLSA)

**DOI:** 10.3389/fpsyt.2020.586260

**Published:** 2020-12-16

**Authors:** Louise Moodie, Gabriela Ilie, Robert Rutledge, Pantelis Andreou, Susan Kirkland

**Affiliations:** ^1^Department of Community Health and Epidemiology, Dalhousie University, Halifax Regional Municipality, NS, Canada; ^2^Department of Urology, Dalhousie University, Halifax Regional Municipality, NS, Canada; ^3^Department of Radiation Oncology, Dalhousie University, Halifax Regional Municipality, NS, Canada; ^4^Department of Psychology and Neuroscience, Dalhousie University, Halifax Regional Municipality, NS, Canada; ^5^Geriatric Medicine, Department of Medicine, Dalhousie University, Halifax Regional Municipality, NS, Canada

**Keywords:** prostate cancer, cancer survivorship, mental health, depression, anxiety, substance use, multimorbidity, quality of life

## Abstract

**Background:** Small-scale studies indicate an increase in mental health disorders among prostate cancer survivors compared to the general population, but large population-based data assessing this relationship are scarce. The present study examined the prevalence of lifetime history of prostate cancer in a cross-sectional sample of Canadian men and assessed the contribution of lifetime history of a prostate cancer diagnosis, multimorbidity, and current alcohol and smoking status to the association with current mental health outcomes in this population.

**Methods:** The analytical sample included 25,183 men (aged 45 to 85 years old), who completed a survey as part of the Canadian Longitudinal Study on Aging (CLSA). The Center for Epidemiological Studies Depression Scale (CES-D10), Kessler's Psychological Distress Scale (K10), and self-reported mental health were mental health outcomes. Multiple logistic regression analyses, and controlling for the complexity of the design and covariates, evaluated the association between prostate cancer survivorship, multimorbidity, alcohol and smoking status, and current mental health outcomes.

**Results:** The prevalence of lifetime history of prostate cancer diagnosis in this population-based sample of men was 4% (95% CI: 3.7, 4.4). Our results indicate statistically significantly higher odds of current psychological distress (aOR = 1.52, 95% CI: 1.09, 2.11) and screening positive for depression (aOR = 1.24; 95% CI: 1.02, 1.51) among survivors of prostate cancer, compared to men without a history of prostate cancer diagnosis in demographics controlled analyses. After addition of multimorbidity and substance use, the odds of screening positive for depression among survivors of prostate cancer are 1.32 (95% CI: 1.06, 1.64) higher compared to men who never had a history of prostate cancer diagnosis.

**Interpretation:** Patient education and empowerment programs aimed at addressing concerns during the diagnosis and treatment and enhancing survivorship care plans by adding routine screening for mental distress to help survivors overcome poor mental health during the cancer survivorship journey, are warranted.

## Introduction

Prostate cancer (PCa) is the most commonly diagnosed cancer among men in Canada, Europe, and the USA (1, 2). Although the incidence rate of PCa increases faster with age than any other cancer, about 81% of PCa diagnosed cases are diagnosed in the early stages of development when patients can receive effective curative treatments (3). While treatment for high-risk PCa patients, especially for healthy younger men, usually is treated with open surgery, laparoscopic or robotic-assisted laparoscopic surgery, which includes the removal of the prostate gland, moderate to low-risk PCa may be treated with radiation and hormonal therapy (2, 3). These treatments have improved considerably over the decades and are providing very good curative results (2–4). However, patients are still at risk of complications and side effects, especially urinary incontinence and erectile dysfunction (4). When these side effects occur among younger patients (e.g., 40 to 65 years of age), they violate expectations regarding urinary and erectile function age norms and often negatively impact the psychological well-being of patients (5–7).

Given that the majority of PCa patients become long-term survivors (>5 years), with over 70% of patients expected to live 10 years or more from the time of diagnosis, unaddressed mental health disorders can negatively impact their health (2–5). According to a literature review conducted by Fervaha et al. (6), one in six men with a PCa diagnosis will experience clinically significant depression, which was shown to contribute to poorer oncological outcomes (6–10). A meta-analysis by Watts and colleagues found that rates of depression and anxiety among PCa survivors were higher than those found in the general population, although most of the studies included in the review had small sample sizes (7). These results have been further corroborated by a population-based study of 6,685 men residing in Atlantic Canada (5). Survivors of PCa had more than double the odds of screening positive for depression and anxiety, compared to men who had no history of cancer (control), while men with a history of any other form of cancer had comparable mental health outcomes with the control group (5).

The rise in PCa incidence with increased age combined with low PCa mortality have important implications for the Canadian healthcare system (11–13). Men with co-occurring depression and PCa are more likely to have worse quality of life, short and long term, increased risk of multimorbidity (e.g., cardiovascular disease and diabetes), increased mortality, and higher healthcare utilization than men in the general population (9, 10). Hence, examining and understanding the mechanisms behind the association between mental health outcomes and PCa survivorship is a health priority (1, 5–7, 10–12).

The mechanisms through which PCa survivorship and mental health are associated have not been directly assessed. However, research shows that mental health disorders have long been associated with multimorbidity and unhealthy lifestyle coping mechanisms, such as alcohol use and smoking (12–17). As 99% of patients diagnosed with PCa are over age 50, and about 65% are over 65 years of age, they are more likely to experience the sequelae of cancer treatment in the context of other coexisting medical conditions (2, 3, 8, 13). Studies show that individuals with multimorbidity have worse physical, social, and psychological quality of life (14). Read et al. (15) found that depression is two to three times more likely to be experienced by people with multimorbidity than those who have no chronic physical conditions (15). Alcohol use and smoking are known unhealthy coping mechanisms and risk factors associated with PCa (16–19). Continued alcohol use and smoking after diagnosis can complicate treatment, increase risk for further malignancy, and contribute to secondary health problems such as cardiovascular disease and diabetes for PCa survivors (16, 18, 19).

In the past few years, we have seen an increase in the number of studies that are beginning to assess the role of psychosocial factors on the mental health and quality of life of prostate cancer survivors (11, 20). For example, a study looking at prostate cancer survivorship among married men (or currently in a relationship) who had undergone surgery for their prostate cancer diagnosis, found that 16.2% of the men in the sample also screened positive for psychological distress (20). This outcome was worse for men who were of younger age, those who scored low on relationship satisfaction, and were not, at the time the study was conducted, on prescribed medication for anxiety, depression, or both (20).

One gap in the literature is the assessment of the contribution of multimorbidity, smoking, and alcohol use in addition to the presence or absence of the lifetime history of a PCa diagnosis on current mental health disorders. A second gap is the absence of an assessment of the prevalence of the presence of a lifetime history of a PCa diagnosis among men in Canada. Last, data on the association between history of PCa and current mental health disorders in Canada are scarce (5). Here we examine the prevalence of PCa and its demographic characteristics and examine the relationship between status of lifetime history of PCa diagnosis and current mental health outcomes, with and without the contribution of multimorbidity, alcohol use and smoking status, in demographics controlled analyses, in a large population-based sample of adult Canadian men who participated in the baseline data collection cycle of the Canadian Longitudinal Study on Aging (CLSA) between 2010 and 2015.

## Methods

### Participants

Data were based on a sample of 25,183 men (45 to 85 years of age) from the slightly more than 50,000 men and women across Canada's 10 provinces who participated in either the Tracking or Comprehensive cohorts of the baseline cycle (2010 to 2015) of the CLSA. The CLSA is a national, longitudinal research platform that collects comprehensive data and biological samples, which support a wide variety of aging-related research. A total of 10,406 individuals were assessed during a telephone interview (Tracking cohort), and 14,777 individuals were assessed in an in-home interview (Comprehensive cohort). Variables in the analyses were assessed in both cohorts, with the exception of psychological distress, which was assessed in the Comprehensive cohort only. Details about CLSA design, recruitment, study procedure, and measures have been described elsewhere (21). The CLSA Protocol is also available for download under the Researchers section of the CLSA website (www.clsa-elcv.ca). All procedures performed in this study were in accordance with the ethical standards of the institutional ethics committee (Dalhousie University; Project 2018-4523). The survey was conducted according to the ethics standards and principles of the *World Medical Association Declaration of Helsinki* guidelines for human studies. All participants have provided written informed consent prior to participating in the study.

### Study Design

#### Outcome Variables

Two validated and one self-report measures were used to assess current mental health.

*Psychological distress* was assessed using Kessler's Psychological Distress Scale (K10) (22). This commonly-used 10-item scale has high levels of internal consistency (Cronbach's alpha = 0.88) and convergent validity (0.84) (22, 23). K10 screens for symptoms of psychological distress, such as depression and anxiety, within the past month (23). Scores were on a K10 range between 10 and 50, with scores under 20 indicating good mental health, 20–24 indicating mild mental disorder, 25–29 indicating moderate mental disorder, and 30 and above indicating the likelihood of a severe mental disorder (22, 23). The cutoff score for psychological distress recommended by Kessler is a score greater than or equal to 20 (22). Screening positive for distress (score ≥ 20) was coded 1, and screening negative for distress (score < 20) was coded 0.

Screening positive for *depression* was assessed using the Center for Epidemiologic Studies—Depression (CES-D10) Scale, which identifies current (past week) symptoms of depression among community-residing older adults (24). The CES-D10 includes 10 items comprising six scales reflecting major facets of depression: depressed mood, feelings of guilt and worthlessness, feelings of helplessness and hopelessness, psychomotor retardation, loss of appetite, and sleep disturbance. The cutoff score for depressive symptoms recommended by Andresen et al. (24) is a score greater than or equal to 10 (24). Screening positive for depressive symptoms (score ≥ 10) was coded 1, and screening negative for depressive symptoms (score < 10) was coded 0. Internal consistency, test–retest reliability, convergent validity and divergent validity for CES-D10 are 0.86, 0.85, 0.91, and 0.89, respectively, and they are used regularly in psychiatric settings as well as general population mental health surveillance research (25–27).

*Self-rated mental health* (SRMH) was assessed through the following question: “*In general, would you say your mental health is excellent, very good, good, fair or poor?”* Research suggests that this question is a good proxy for clinical criteria for mental health assessment (28). Specifically, paralleling the literature on clinical mental health assessment, research results highlight a positive association between “poor” self-perceived mental health status, depressive symptoms, and mental health treatment (28, 29). The Likert scale question surveyed assessed the presence of poor mental health (e.g., fair and poor) and absence of poor mental health (e.g., excellent, very good, and good), coded 1 and 0, respectively. This dichotomization is commonly seen in the literature for self-rated mental health (29).

#### Exposure Variables

*Lifetime History of a PCa Diagnosis* was the main predictor. CLSA participants were asked: “*Has a doctor ever told you that you had cancer?*” Participants who answered “yes” were further asked “*What type(s) of cancer were you diagnosed with?*” Lifetime history of PCa diagnosis was coded 1, and absence of lifetime history of PCa diagnosis was coded 0.

#### Multimorbidity

Participants were asked if they had any one of the following 26 physical health conditions: heart attack or myocardial infarction; angina (or chest pain due to heart disease); stroke or CVA (cerebrovascular accident); high blood pressure or hypertension; heart disease (including congestive heart failure); peripheral vascular disease or poor circulation in limbs and mini-stroke or TIA (Transient Ischemic Attack); diabetes, borderline diabetes, or high blood sugar; overactive thyroid (hyperthyroidism); underactive thyroid (hypothyroidism); emphysema, chronic bronchitis, chronic obstructive pulmonary disease (COPD), or chronic changes in lungs due to smoking; asthma; macular degeneration; osteoarthritis in one or both hands, osteoarthritis in one or both hips, osteoarthritis in the knee; osteoporosis; other type of arthritis; rheumatoid arthritis; kidney disease or kidney failure; intestinal or stomach ulcers; bowel disorder; epilepsy, multiple sclerosis, and Parkinsonism/Parkinson's disease; dementia or Alzheimer's disease; and cancer.

These chronic conditions have been included in previous studies assessing multimorbidity (27–29). Consistent with previous research, we defined multimorbidity as the presence of two or more chronic physical health conditions (coded 1; less than two chronic conditions coded 0).

#### Smoking

Participants were asked about the frequency of smoking cigarettes in the past 30 days. Responses indicated daily, occasional (at least one cigarette in the past 30 days, but not every day) and no cigarette smoking at all, and were coded 2, 1, and 0, respectively. This item is used in the Ontario Health Study (30), as well as the Canadian Health Measures Survey (CHMS) (31) and the Canadian Tobacco Use Monitoring Survey (CTUMS) (32), all current and reliable sources of population health surveillance with an established epidemiological and peer reviewed scientific reputation.

#### Alcohol Use

Participants were asked about the frequency of their alcohol consumption in the past 12 months. Responses included daily drinking (four to five times a week or almost every day), weekly drinking (once to two to three times a week), occasional drinking (either less than once a month, about once a month or two to three times a month), and no alcohol drinking at all, coded 3, 2, 1, and 0, respectively. This item was adapted from the Ontario Health Survey (30), which were originally sourced from the Center for Addiction and Mental Health Monitor (33), one of the longest and most reputable surveys on adult mental health in the world.

### Covariates

Covariates were selected based on previous literature indicating that these variables are associated with mental health (34–40). The following six covariates were employed: age (<65, 65–74, >74), province (Alberta, British Columbia, Manitoba, New Brunswick, Newfoundland and Labrador, Nova Scotia, Ontario, Prince Edward Island, Quebec, and Saskatchewan), education (less than post-secondary degree/diploma and post-secondary degree/diploma), household income (<$50,000 a year; ≥$50,000, <$100,000 a year; ≥$100,000, <$150,000 a year; ≥$150,000 a year), marital status (married or common law; not married/not common-law: single, never married, never lived with partner, widowed, divorced, or separated), and ethnicity [other (non-white), white only, to account for less than five cell counts].

### Statistical Analysis

Data derived from complex surveys using stratification and weights do not meet the assumption of independent observations and thus underestimate variances, overstating significance levels, which may result in false-positive inferences. We therefore employed design-based estimation methods to accommodate these sampling methods (41). Following the CLSA guidelines, trimmed (inflation) weights (in the descriptive analyses) and analytic weights (in the regression analyses) were used in order for the estimates to be generalizable to the Canadian population (42). To adjust for the complex survey data, variances were estimated using Taylor Series Linearization available in the Complex Sample module in SPSS 25.0. For the pooled (Tracking and Comprehensive cohorts) data, our analyses were based on a design employing normalized inclusion weights of 25,183 adult men drawn from 34 equally allocated strata. For the Comprehensive CLSA data, our analyses were based on a design employing normalized inclusion weights of 14,777 adult men drawn from 14 equally allocated strata. Covariate-adjusted logistic regressions assessed the association between lifetime history of a PCa diagnosis and three mental health outcomes—psychological distress, depressive symptoms, and self-rated mental health, in covariate-adjusted analyses. Covariate-adjusted multiple logistic regressions also evaluated the contribution of lifetime history of PCa, multimorbidity, and substance use (alcohol use and smoking) in predicting each of the three mental health outcomes, in covariate-adjusted analyses. Listwise deletion reduced the estimation sample for depressive symptoms to 25,060 from 25,183, and for self-rated mental health to 25,159 from 25,183. For psychological distress, listwise deletion reduced the estimation sample to 13,960 from 14,777. Based on 5.5% of our data missing for psychological distress, multiple imputation (MI) was performed using IBM SPSS 25 (43). The imputation method selected was the Automatic method (the software scans the data to determine which method of imputation (monotone or fully conditional) should be specified, based on the pattern of missing data. Fully conditional specification (FCS) was determined, which is an iterative Markov Chain Monte Carlo (MCMC) method. The MCMC method can be used when the pattern of missing data is arbitrary (44). We specified 20 iterations, as recommended by the literature (44, 45). For each iteration, and for each variable in the order specified in the variable list, the FCS method fits a univariate model using all other available variables in the model as predictors, then imputes missing values for the variable being fit. This method continues until the maximum number of iterations is reached. For each of our analyses using MI, sensitivity analyses were performed to determine how the missing data affected the results (45). Results of the MI analyses can be found in the (Supplementary Tables 1a, 2a).

## Results

An estimated 4% of adult men (95% CI: 3.7, 4.4) reported a lifetime history of PCa diagnosis. Table 1 displays the estimates of lifetime history of PCa diagnosis by demographic characteristics. Of the Canadian men who reported a lifetime history of a PCa diagnosis, 72.8% were over the age of 65. The majority of men who reported a lifetime history of PCa diagnosis were married or common-law (83.7%). Additionally, over 70% of the men who reported a history of PCa had a household income of <$100,000 per year, and of those, 29% reported a household income of <$50,000 per year. The results indicate higher odds (OR = 1.20; 95% CI: 1.04, 1.39) of lifetime history of PCa among married men compared to single men, and among men older than 65 years old (OR = 5.03, 95% CI: 4.27, 5.93, and OR = 9.50 95% CI: 8.11, 11.07 times higher for men between 65 and 75 years old, or older than 75, respectively) compared to men younger than 65 years old. Increased total household income and education were protective factors for lifetime history of PCa. Odds for lifetime history of PCa were almost double (OR = 1.80; 95% CI: 1.23, 2.65) among PEI residents compared to Alberta residents (reference category), although, overall, province of residence was not statistically significantly associated with lifetime history of PCa diagnosis status.

**Table 1:** Weighted and unweighted estimates and odds-rations (ORs) of lifetime history of prostate cancer (PCa) diagnosis by demographic characteristics for Canadian men from the baseline cycle (2010–2015) of the CLSA (*N* = 25,183).

	**Lifetime history of PCa diagnosis**	
	**No (*n* = 23,825) %^**a**^ (95% CI) OR^**b**^ (95% CI)**	**Yes (*n* = 1,358) %^**a**^ (95% CI) OR^**b**^ (95% CI)**
Age^1^	Wald F (2, 25,148) = 403.52^***^	
>74 (*n*)	(4,133)	(641)
Weighted^a^	87.1 (85.5, 88.5)	12.9 (11.5, 14.5)
Unweighted	86.6 (86.2, 87.0)	13.4 (11.6, 15.2)
	1.00 (Reference)	9.50 (8.11, 11.07)^***^
65–74 (*n*)	(5,516)	(452)
Weighted	92.3 (91.0, 93.4)	7.7 (6.6, 9)
Unweighted	92.4 (92.1, 92.7)	7.6 (6.2, 9.0)
	1.00 (Reference)	5.03 (4.27, 5.93)^***^
<65 (*n*) (Reference)	(14,176)	(265)
Weighted	98.4 (98.1, 98.7)	1.6 (1.3, 1.9)
Unweighted	98.2 (98.0, 98.4)	1.8 (1.1, 2.5)
Provincial Residence^2^	Wald F (9, 25,141) = 1.69	
Saskatchewan (*n*)	(641)	(38)
Weighted	95.7 (94.1, 96.9)	4.3 (3.1, 5.9)
Unweighted	94.4 (94.1 94.7)	5.6 (4.4, 6.8)
	1.00 (Reference)	1.21 (0.82, 1.80)
Quebec (*n*)	(4,465)	(223)
Weighted	96.2 (95.5, 96.8)	3.8 (3.2, 4.5)
Unweighted	95.2 (94.9, 95.5)	4.8 (3.7, 5.9)
	1.00 (Reference)	1.06 (0.83, 1.37)
Prince Edward Island (*n*)	(517)	(48)
Weighted	93.7 (91.5, 95.4)	6.3 (4.6, 8.3)
Unweighted	91.5 (91.1, 91.9)	8.5 (7.0, 10.0)
	1.00 (Reference)	1.80 (1.23, 2.65)^***^
Ontario (*n*)	(5,210)	(298)
Weighted	95.9 (95.1, 96.5)	4.1 (3.5, 4.9)
Unweighted	94.6 (94.3, 94.9)	5.4 (4.2, 6.6)
	1.00 (Reference)	1.10 (0.87, 1.40)
Nova Scotia (*n*)	(2,177)	(129)
Weighted	96.4 (95.3, 97.3)	3.6 (2.7, 4.7)
Unweighted	94.4 (94.1, 94.7)	5.6 (4.4, 6.8)
	1.00 (Reference)	1.20 (0.91, 1.59)
Newfoundland and Labrador (*n*)	(1,622)	(85)
Weighted	96.1 (94.6, 97.2)	3.9 (2.8, 5.4)
Unweighted	95.0 (94.7, 95.3)	5.0 (3.8, 6.2)
	1.00 (Reference)	1.00 (0.73, 1.37)
New Brunswick (*n*)	(634)	(34)
Weighted	96.3 (94.7, 97.4)	3.7 (2.6, 5.3)
Unweighted	94.9 (94.6, 95.2)	5.1 (3.9, 6.3)
	1.00 (Reference)	1.04 (0.69, 1.59)
Manitoba (*n*)	(2,137)	(107)
Weighted	96.3 (95.4, 97)	3.7 (3, 4.6)
Unweighted	95.2 (94.9, 95.5)	4.8 (3.7, 5.9)
	1.00 (Reference)	0.95 (0.72, 1.27)
British Columbia (*n*)	(4,089)	(264)
Weighted	95.6 (94.8, 96.3)	4.4 (3.7, 5.2)
Unweighted	93.9 (93.6, 94.2)	6.1 (4.8, 7.4)
	1.00 (Reference)	1.21 (0.94, 1.54)
Alberta (*n*) (Reference)	(2,333)	(132)
Weighted	96.2 (95.3, 97)	3.8 (3, 4.7)
Unweighted	94.6 (94.3, 94.9)	5.4 (4.2, 6.6)
Education^3^	Wald F (1, 25,074) = 26.22^***^	
Post-secondary degree/diploma (*n*)	(18,032)	(955)
Weighted	96.3 (95.9, 96.7)	3.7 (3.3, 4.1)
Unweighted	95.0 (94.7, 95.3)	5.0 (3.8, 6.2)
	1.00 (Reference)	0.71 (0.62, 0.81)^***^
Less than post-secondary degree/diploma (*n*) (Reference)	(5,721)	(400)
Weighted	95.1 (94.2, 95.8)	4.9 (4.2, 5.8)
Unweighted	93.5 (93.2, 93.8)	6.5 (5.2, 7.8)
Total Household Income^4^	Wald F (3, 23,986) = 25.07^***^	
≥$150,000 year (*n*)	(3,987)	(136)
Weighted	97.7 (96.9, 98.3)	2.3 (1.7, 3.1)
Unweighted	96.7 (96.5, 97.0)	3.3 (2.3, 4.3)
	1.00 (Reference)	0.44 (0.36, 0.56)^***^
≥$100,000, <$150,000/year (*n*)	(4,644)	(221)
Weighted	96.5 (95.6, 97.2)	3.5 (2.8, 4.4)
Unweighted	95.5 (95.2, 95.8)	4.5 (3.4, 5.6)
	1.00 (Reference)	0.64 (0.53, 0.77)^***^
≥$50,000, <$100,000/year (*n*)	(8,356)	(561)
Weighted	95.6 (94.9, 96.2)	4.4 (3.8, 5.1)
Unweighted	93.7 (93.4, 94.0)	6.3 (5.0, 7.6)
	1.00 (Reference)	0.98 (0.84, 1.12)^**^
<$50,000/year (n) (Reference)	(5,744)	(373)
Weighted	95.1 (94.2, 95.8)	4.9 (4.2, 5.8)
Unweighted	93.9 (93.6, 94.2)	6.1 (4.8, 7.4)
Marital Status^5^	Wald F (1, 25,143) = 6.05^*^	
Married/common-law (*n*)	(18,454)	(1,084)
Weighted	95.9 (95.4, 96.3)	4.1 (3.7, 4.6)
Unweighted	94.5 (94.2, 94.8)	5.5 (4.3, 6.7)
	1.00 (Reference)	1.20 (1.04, 1.39)^*^
Non-married/non-common-law (*n*) (Reference)	(5,365)	(274)
Weighted	96.5 (95.8, 97.1)	3.5 (2.9, 4.2)
Unweighted	95.1 (94.8, 95.4)	4.9 (3.8, 6.0)
Ethnicity^6^	Wald F (1, 24,084) = 0.71	
White only (*n*)	(21,800)	(1,273)
Weighted	95.9 (95.5, 96.2)	4.1 (3.8, 4.5)
Unweighted	94.5 (94.2, 94.8)	5.5 (4.3, 6.7)
	1.00 (Reference)	1.14 (0.84, 1.56)
Other (*n*) (Reference)	(992)	(53)
Weighted	96.7 (94.7, 98.0)	3.3 (2.0, 5.3)
Unweighted	94.9 (94.6, 95.2)	5.1 (3.9, 6.3)

1,2 *No missing values: n = 25,183*.

3 *Listwise deletion n = 25,108*.

4 *Listwise deletion n = 24,022*.

5 *Listwise deletion resulted in the following: n = 25,117*.

6 *Listwise deletion resulted in the following: n = 24,118*.

*In complex samples, df = (#PSUs)–(# strata)*.

*** *Significant at p < 0.001*.

** *Significant at p < 0.01*.

* *Significant at p < 0.05*.

a *Complex sample analysis design adjusted using inflation weights*.

b *Complex sample analysis design adjusted using analytical weights*.

Logistic regression analyses, adjusted for covariates and the complexity of the design, revealed that men with a lifetime history of PCa diagnosis had statistically significantly higher adjusted odds of screening positive for psychological distress (aOR = 1.52, 95% CI: 1.09, 2.11; multiple imputation aOR = 1.57, 95% CI: 1.18, 2.08), depression (aOR = 1.24, 95% CI: 1.02, 1.51), but not self-rated mental health (aOR = 0.95, 95% CI: 0.68,1.32), compared to men who never received a PCa diagnosis (Table 2). Multiple imputation results for psychological distress analyses can be found in Supplementary Table 1a.

**Table 2:** Weighted estimates and logistic regression analyses examining the association between lifetime history of PCa and mental health assessed through psychological distress (K10), depression (CES-D 10), and poor self-rated mental health (SRMH) for Canadian men from the baseline cycle of the CLSA, 2010–2015.

	**Screened positive for psychological distress (K10) (*****N*** **= 13,960)**^1^	
	**No (*n* = 12,623)**	**Yes (*n* = 1,337)**
	F (7, 12,689) = 39.29^***^	
Lifetime history of PCa diagnosis	F (1, 12,695) = 6.24^*^	
Yes (*n*)	(681)	(79)
aOR^a^ (95% CI)	1.00 (Reference)	1.52 (1.09, 2.11)^**^
No (*n*) (Reference)	(11,942)	(1,258)
	**Screened positive for depression (CES-D 10)** (*N* = 25,060)	
	**No** (*n* = 21,750)	**Yes** (*n* = 3,310)
	F (7, 22,777) = 94.98^***^	
Lifetime history of PCa diagnosis	F (1, 22,783) = 4.51^*^	
Yes (n)	(20,582)	(3,125)
aOR^a^ (95% CI)	1.00 (Reference)	1.24 (1.02, 1.51)^*^
No (*n*) (Reference)	(1,168)	(185)
	**Poor self-rated mental health (SRMH)** (*N* = 25,159)	
	No (*n* = 22,564)	Yes (*n* = 1,238)
	F (7, 22,860) = 53.87^***^	
Lifetime history of PCa diagnosis	F *(1, 22,866) = 0.11*	
Yes (*n*)	(1,302)	(1,302)
aOR^a^ (95% CI)	1.00 (Reference)	0.95 (0.68, 1.32)
No (*n*) (Reference)	(22,564)	(1,238)

*** *Significant at P < 0.001*.

** *Significant at P < 0.01*.

* *Significant at P < 0.05 (two-tailed)*.

1 *Comprehensive cohort only*.

a *Analyses were controlled for age, province, education, household income, marital status, ethnicity, and complexity of the design*.

Multiple logistic regression analyses, adjusted for the covariates and the complexity of the design, examined the contribution of lifetime history of PCa diagnosis, multimorbidity, smoking, and alcohol use on each of the three mental health outcomes, and revealed a statistically significant contribution of all four predictors to depression, but not psychological distress or self-rated mental health (Table 3). Odds were 1.32 (95% CI: 1.06, 1.64) higher for screening positive for depressive symptoms among men with a lifetime history of PCa compared with men without a lifetime history of PCa, and were 1.62 (95% CI: 1.45, 1.80) higher for screening positive for depression among men with multimorbidity than those without. Men who were weekly or daily drinkers had odds 1.18 (95% CI: 1.02, 1.35) and 1.47 (95% CI: 1.25, 1.73) times significantly higher for screening positive for depression than those who were non-drinkers, respectively. Daily smokers also had 1.57 (95% CI: 1.36, 1.82) times higher odds for screening positive for depression at the time they were being surveyed compared to men who were non-smokers.

**Table 3:** Multivariate logistic regression results predicting current psychological distress (K10), depression (CES-D 10), and self-rated poor mental health, by status of lifetime history of PCa, multimorbidity, alcohol use, and smoking for Canadian men from the baseline cycle of the CLSA, 2010–2015.

	**Screening positive for psychological distress vs. screening negative^1^** ***% (95% CI)*** ** aOR^**a**^ (95% CI)**	**Screening positive for depression vs. screening negative ** ***% (95% CI)*** ** aOR^**a**^ (95% CI)**	**Poor self-rated mental health vs. good self-rated mental health ** ***% (95% CI)*** ** aOR ^**a**^ (95% CI)**
	F (21, 8,989) = 15.42^***^	F (24, 16,477) = 28.98^***^	F (24, 16,542) = 19.97^***^
Lifetime history of PCa diagnosis	F *(1,9,009) = 1.83*	F (1, 16,500) = 6.03^*^	F (*1, 16,565) = 0.002*
Yes	*10.2 (7.5, 13.7)* 1.30 (0.89, 1.89)	*15.1 (12.7, 17.8)* 1.32 (1.06, 1.64)^*^	*4.3 (3.1, 5.9)* 1.01 (0.70, 1.45)
No	1.00 (Reference)	1.00 (Reference)	1.00 (Reference)
Multimorbidity	F (1,9,009) = 31.29^***^	F (1, 16,500) = 75.29^***^	F (1, 16,565) = 62.19^***^
Yes	12.5 (11.4, 13.6) 1.64 (1.38, 1.95)^***^	16.7 (15.9, 17.6) 1.62 (1.45, 1.80)^***^	7.2 (6.6, 7.8) 1.97 (1.67, 2.33)^***^
No	1.00 (Reference)	1.00 (Reference)	1.00 (Reference)
Alcohol use	F (3,9,007) = 6.14^**^	F (3, 16,498) = 13.79^***^	F (3, 16,563) = 8.76^***^
Daily drinker	13.6 (11.8, 15.5) 1.59 (1.20, 2.09)^**^	16.5 (15.2, 17.8) 1.47 (1.25, 1.73)^**^	6.3 (5.5, 7.2) 1.58 (1.25, 2.01)^**^
Weekly drinker	9.2 (8.0, 10.5) 1.52 (1.21, 1.91)^*^	12.0 (11.1, 13.0) 1.18 (1.02, 1.35)^*^	4.6 (4.0, 5.3) 1.06 (0.85, 1.32)
Occasional drinker	8.2 (7.2, 1.59) 1.12 (0.91, 1.39)	11.0 (10.1, 12.00) 0.88 (0.77, 1.01)	4.1 (3.5, 4.7) 0.84 (0.68, 1.05)
Non-drinker	1.00 (Reference)	1.00 (Reference)	1.00 (Reference)
Smoking	F (2,9,008) = 12.91^***^	F (2, 16,499) = 18.23^***^	F (2, 16,564) = 9.36^***^
Daily smoker	20.4 (17.2, 24.0) 1.84 (1.435 2.34)^***^	23.9 (21.7, 26.3) 1.57 (1.36, 1.82)^***^	10.5 (9.0, 12.3) 1.54 (1.25, 1.89)^***^
Occasional smoker	14.3 (9.5, 21.1) 1.41 (0.86, 2.32)	16.5 (13.0, 20.9) 1.21 (0.91, 1.64)	8.3 (5.8, 11.7) 1.50 (0.98, 2.29)
Non-smoker	1.00 (Reference)	1.00 (Reference)	1.00 (Reference)
Age	F (2,9,008) = 35.91^***^	F (2, 16,499) = 51.79^***^	F (2, 16,564) = 8.76^***^
<65	*11.4 (10.5, 12.5)* 2.31 (1.80, 2.95)^***^	*14.6 (13.9, 15.4)* 1.79 (1.55, 2.07)^***^	*6.2 (5.7, 6.8)* 2.40 (1.91, 3.00)^***^
65–74	7.5 % (6.5, 8.7) 1.14 (0.87, 1.47)	11.2 (10.2, 12.2) 1.01 (0.86, 1.17)	3.3 (2.8, 3.9) 0.88 (0.68, 1.14
>74	1.0 (Reference)	1.0 (Reference)	1.0 (Reference)
Province	F (6,9,004) = 6.98^***^	F (9, 16,492) = 1.92^*^	F (9, 16,557) = 4.75^***^
Saskatchewan	–	*15.7 (12.5, 19.6)* 1.05 (0.76, 1.46)	*5.7 (3.8, 8.4)* 0.75 0(.45, 1.24)
Quebec	*14.6 (12.9, 16.6)* 1.23 (0.88, 1.73)	*13.7 (12.4, 15.0)* 0.87 (0.70, 1.08)	*3.8 (3.1, 4.5)* 0.46 (0.33, 0.65)^***^
Prince Edward Island	–	*13.5 (10.1, 17.7)* 0.83 (0.58, 1.22)	*5.8 (3.7, 8.8)* 0.72 (0.42, 1.24)
Ontario	*8.9 (7.5, 10.4)* 0.79 (0.56, 1.12)	*12.9 (11.8, 14.2)* 0.95 (0.77, 1.18)	*5.9 (5.1, 6.9)* 0.93 (0.68, 1.28)
Nova Scotia	*7.4 (5.7, 9.5)* 0.58 (0.38, 0.88)^*^	*13.9 (12.0, 15.9)* 0.93 (0.73, 1.18)	*6.2 (5.0, 7.6)* 0.84 (0.58, 1.21)
Newfoundland and Labrador	*6.0 (4.3, 8.3)* 0.47 (0.30, 0.75)^**^	*11.2 (9.3, 13.4)* 0.76 (0.58, 0.99)^*^	*4.4 (3.2, 5.9)* 0.60 (0.39, 0.92)^*^
New Brunswick	–	*14.8 (11.7, 18.5)* 0.90 (0.65, 1.25)	*5.2 (3.4, 7.9)* 0.63 (0.37, 1.05)
Manitoba	*8.9 (6.8, 11.4)* 0.72(0.48, 1.09)	*14.9 (12.9, 17.2)* 1.06 (0.83, 1.36)	*5.8 (4.5, 7.4)* 0.83 (0.56, 1.23)
British Columbia	*10.8 (9.2, 12.7)* 0.97 (0.69, 1.38)	*15.2 (13.8, 16.8)* 1.13 (0.91, 1.40)	*6.2 (5.3, 7.3)* 0.93 (0.66, 1.30)
Alberta	1.0 Reference	1.0 Reference	1.0 Reference
Education	F *(1,9,009) = 0.34*	F *(1, 16,500) = 2.43*	F *(1, 16,565) = 0.69*
Less than post-secondary degree/diploma	*12.7 (11.0, 14.6)* 1.06 (0.87, 1.29)	*17.1 (15.8, 18.4)* 1.10 (0.98, 1.23)	*6.5 (5.7, 7.4)* 0.93 (0.78, 1.11)
Post-secondary diploma	1.0 Reference	1.0 Reference	1.0 Reference
Household income	F (3,9,007) = 13.08^***^	F (3, 16,498) = 37.31^***^	F (3, 16,563) = 26.20^***^
<$50,000/year	*18.1 (16.1, 20.2)* 2.56 (1.87, 3.50)^***^	*22.9 (21.6, 24.4)* 2.65 (2.16, 3.24)^***^	*9.9 (8.9, 10.9)* 3.48 (2.59, 4.86)^***^
≥$50 000, <$100,000 per year	*10.9 (9.6, 12.2)* 1.74 (1.31, 2.31)^***^	*12.9 (11.9, 13.8)* 1.61 (1.33, 1.94)^***^	*5.4 (4.7, 6.0)* 2.12 (1.55, 2.89)^***^
≥$100,000, <$150 000 per year	*8.3 (6.9, 9.9)* 1.36 (1.00, 1.86)^*^	*11.1 (9.9, 12.4)* 1.44 (1.76, 1.77)^***^	*3.3 (2.7, 4.2)* 1.30 (0.90, 1.86)
≥$150,000 year	1.0 Reference	1.0 Reference	1.0 Reference
Marital status	F (1,9,009) = 11.52^*^	F (1, 16,500) = 85.17^***^	F (1, 16,565) = 42.98^***^
Non-married/non-common-law	*17.7 (15.7, 19.9)* 1.41 (1.16, 1.72)^**^	*24.4 (22.8, 26.0)* 1.75 (1.56, 1.98)^***^	*11.1 (9.9, 12.3)* 1.82 (1.52, 2.18)^***^
Married/common-law	1.0 Reference	1.0 Reference	1.0 Reference
Ethnicity	F *(1,9,009) = 0.85*	F *(1, 16,500) = 3.16*	F *(1, 16,565) = 2.98*
Other	*14.4 (10.7, 19.2)* 1.20 (0.82, 1.76)	*17.8 (14.4, 21.8)* *1.28 (0.98, 1.67)*	*4.3 (2.8, 6.6)* *0.66 (0.41, 1.06)*
White only	1.0 Reference	1.0 Reference	1.0 Reference

*** *Significant at P < 0.001*.

** *Significant at P < 0.01*.

* Significant at P < 0.05 (two-tailed).

1 *Comprehensive cohort only*.

a *Controlled for age, province, education, household income, marital status and ethnicity, and the complexity of the design*.

Lifetime history of PCa was not a statistically significant contributor of screening positive for psychological distress (aOR = 1.30; 95% CI: 0.89, 1.89), while MI analyses (see Supplementary Table 2a) show that among men with a lifetime history of PCa, odds were 1.62 (95% CI: 1.22, 2.16) significantly higher for screening positive for psychological distress compared with men without a history of PCa. Odds were 1.64 (95% CI: 1.38, 1.95; multiple imputation aOR = 1.74, 95% CI: 1.52, 1.98) higher for screening positive for psychological distress among men with multimorbidity than those without. Men who identified themselves as weekly drinkers had 1.52 (95% CI: 1.21, 1.91; multiple imputation aOR = 1.62, 95% CI: 1.32, 1.98) and those who identified themselves as daily drinkers had 1.59 (95% CI: 1.20, 2.09; multiple imputation aOR = 1.44, 95% CI: 1.18, 1.75) greater odds of screening positive for psychological distress than non-drinkers. Men who identified themselves as daily smokers had 1.84 (95% CI: 1.43, 2.34; multiple imputation aOR = 1.82 (95% CI: 1.43, 2.30) times higher odds for screening positive for psychological distress compared to men who identified as non-smokers.

Last, lifetime history of PCa was not a statistically significant contributor of poor self-rated mental health (aOR = 1.01; 95% CI: 0.70, 1.45). Odds were 1.97 (95% CI: 1.67, 2.33) higher for poor self-rated mental health among men with multimorbidity than those without and were 1.58 (95% CI: 1.25, 2.01) times higher for self-rated poor mental health among daily alcohol drinkers compared to men who identified as non-drinkers. Last, men who identified as daily cigarettes smokers had 1.54 (95% CI:1.25, 1.89) times higher odds for self-rated poor mental health, compared to non-cigarettes smokers.

## Discussion

To our knowledge, this is the first study to assess the prevalence of PCa survivorship in Canada. Results indicate that 4% of adult men in this Canadian population-based sample reported having had a lifetime history of PCa diagnosis between 2010 and 2015 when the baseline for CLSA was collected. This estimate is comparable to the 3.9% prevalence estimate of lifetime history of PCa in Atlantic Canada (2009–2015) characterized by similar demographic characteristics (e.g., age, education, ethnicity, household income) (5). Results indicate greater psychological distress and depressive symptoms among Canadian men who reported a lifetime history of PCa diagnosis, compared to men who had no history of a PCa diagnosis in demographics-controlled analyses. To our knowledge, this is the largest population-based study examining these associations. Results corroborate findings from previous smaller sample size investigations and indicate that mental health disorders are significantly prevalent among prostate cancer survivors compared to men who never had a prostate cancer diagnosis, and support evidence that mental health care should be part of prostate cancer survivorship plans (5–7). While a statistically significant association between lifetime history of PCa diagnosis and validated mental health outcomes (psychological distress and depression) was observed, the status of lifetime history of PCa was not associated with self-reported mental health. Adding and holding constant the contribution of multimorbidity, smoking, and alcohol use in covariate-adjusted analyses did not change the association between lifetime history of PCa diagnosis and depression or self-reported mental health, but it rendered the association with psychological distress untenable. A scoping review of the relationship between self-rated mental health and validated measures of mental health (which includes K10) shows that these measures, though related, should not be considered equivalent or interchangeable because self-rated mental health may be measuring people's commonly held perception of mental health and may reflect equating the question to mean whether or not the individual looked for mental health help or resources available in their community (46). Compounding this issue is evidence showing that, compared to women, men tend to report less self-perceived mental health disorders, although the rates of social isolation and suicide are higher among men compared to women (47). These differences may point to the possibility of less communication or verbalization of mental health problems among men, and/or more issues of shame or guilt around acknowledging these problems among men. The present findings caution us to be particularly attentive to symptoms of mental health among men when they are observed. They further emphasize the importance of including validated questionnaires in PCa survivorship plans to assess mental health disorders among PCa during their survivorship journey. The implementation of innovative and integrative patient education and empowerment plans through holistic interventions that aim to ease the psychosocial and physical needs (e.g., loss of sexual function, urinary leakage, feeling disconnected from their intimate partner and close family and friends, lack of sleep, and fatigue) of these survivors is warranted (48, 49). These programs share the common goal of fostering networks of healthcare professionals, researchers, and patient representatives to improve the quality of life of survivors of PCa and intervene early after the diagnosis when most of these survivorship needs emerge (49).

The observed results extend the current literature by showing that PCa survivors may deal with higher levels of depression than men without a lifetime history of PCa diagnosis, even when demographic characteristics and factors known to be associated with poorer mental health outcomes in the general population, such as multimorbidity, alcohol use, and smoking, are statistically controlled (5–10). These results are consistent with results, reported in the literature among smaller sample size populations, that indicate higher odds of depression among survivors of PCa compared to men in the population who do not have a history of PCa (5–7). Results that we report are also consistent with the literature showing that men with higher multimorbidity, or who are frequent current drinkers or smokers, have higher odds for screening positive for mental health disorders compared to those without these conditions (15, 16, 18). Future studies should examine the possibility of interactive effects between these factors and the presence or absence of PCa diagnosis, and their contribution to mental health disorders among men.

Covariate results from our final analysis also indicate that younger age, not being currently in a relationship, lower household income, and residing in Atlantic Canada may be placing men at higher odds of mental health disorder than being of older age, being currently in a relationship, of over 150,000$ in household income and residing in Alberta. These results are not surprising and are comparable to other studies (5, 11, 20, 50, 56).

The results reported here, however, are not without limitations. First, the nature of the data is retrospective and self-reported; thus, it is subject to challenges of accurate recall and also survivor bias, reflecting largely determinants of survival. Since the CLSA data does not capture the date of the PCa diagnosis, survivorship time could not be controlled for in the analyses and may have biased the results. Second, the overall response proportion in the CLSA data was ~10% which (although adjusted for by the use of population-based weights) may have introduced non-response bias (42). CLSA respondents were slightly more educated than the non-responders (21). This bias could have led to an underestimate of the prevalence estimate of history of PCa diagnosis as adults with higher educational attainment tend to live healthier and longer lives than their counterparts (38).

Since multimorbidity and substance use increase the risk of mortality, the proportion of cases with high multimorbidity and heavy substance use may be lower in our data and could have led to an underestimate of the odds ratios observed (51–53). While a binary coding of multimorbidity with a cutoff score of two or more comorbid conditions is common in two thirds of public health studies, possible disease counts, rather than binary coding, may be more appropriate, as suggested by a recent systematic review (54).

Another limitation to our study is the amount of missing data exceeding 5% for the psychological distress (K10) outcome measure, which we addressed through MI and sensitivity analyses. When psychological distress was assessed in a model controlled for demographics, a statistically significant relationship between the predictor and outcome emerged in both original and MI with sensitivity analyses. However, when multimorbidity and substance use were added to the model in the original data set, the association between lifetime history of PCa and psychological distress became non-significant, while in MI data and sensitivity analyses, the results were comparable to the single assessment of the relationship between PCa survivorship and psychological distress, controlled for demographic covariates. The use of a second validated outcome measure (CES-D 10) adds confidence to the MI and sensitivity analyses results for psychological distress, although future studies should attempt to reproduce these results. This study would have benefited from statistical control of current medication for depression, anxiety, or both, the presence of other mental health conditions, or past lifetime history of such conditions. Future studies should consider their inclusion.

Last, our design precludes us from differentiating between men with lifetime history of PCa, men with a lifetime history of any other type of cancer, and men who never had a history of cancer, as our data access file contained a combined comparison (control) group. Future studies may consider undertaking an examination of various other control groups including other types of cancer groups in addition to a no cancer diagnosis control. In a recent study of over 6,000 men residing in Atlantic Canada, it was found that survivors of PCa had more than double the odds of screening positive for anxiety or depression when compared to men from a pooled control group, similar to ours, that included other cancer survivors and men who never had a history of cancer diagnosis combined (5). In a subsequent study, the researchers split their control group and found odds 2.60 times higher for screening positive for depression symptoms among men with a lifetime history of PCa compared to men with a history of any other form of cancer (54). No statistically significant differences emerged when men with other forms of cancer were compared with men who did not report a history of any type of cancer during their lifetime (55). Future studies should consider expanding their control group to other cancer sites. Taken together, these results corroborate the existing literature that mental health disorders are particularly prevalent among PCa survivors and strengthen the recommendation of including mental health assessments in diagnosis to treatment and survivorship care plans for this population (5, 49, 55).

Despite these enumerated limitations, the results reported here provide important contributions to the literature and information for survivorship clinical care teams for this population. Here we provide evidence of poor mental health outcomes among Canadian men with a history of PCa diagnosis by examining CLSA data from a large national population-based survey that used a standard protocol. Another strength to this study is the use of two validated mental health measures, CES-D 10 and K10, in addition to a measure of self-reported mental health, which allowed us to capture different dimensions of the mental health construct. Another important consideration is that we were able to capture 26 chronic conditions for inclusion in our multimorbidity variable. In contrast, many previous studies examining multimorbidity in the Canadian population were based on only a limited number of chronic conditions (56).

Future CLSA longitudinal data should be used to assess temporal changes in the prevalence of mental health outcomes among men with a history of a PCa diagnosis. Future cycles of CLSA data could be used to identify incident cases of PCa, which will also allow for a comparison between the mental health outcomes of newly diagnosed men (incident cases) and those of men with a lifetime history of a PCa diagnosis at baseline. Future studies may also wish to examine the extent to which unhealthy lifestyles are factors that lead to the development of prostate cancer or emerge after the diagnosis as unhealthy coping mechanisms, or both.

In conclusion, data from a nationally representative sample of men indicate that those with a lifetime history of PCa diagnosis have worse mental health outcomes than those with no history of PCa diagnosis, when literature-derived relevant contributing factors to poor mental health outcomes in the population are held constant. These results are important because, taken together with other recent reports in the literature, they point out to a need that is currently unacknowledged and unaddressed in PCa survivorship care plans throughout Canada or elsewhere in the world (5–7, 49, 55). Leaving mental health needs unattended leads to poor quality of life among PCa survivors, poor oncological outcomes, and could potentially burden health care systems (6–11). In light of the current and recent studies, the necessity of assessing patients' needs as they emerge after the diagnosis and treatment, understanding the role treatment modalities play in their development, and creating survivorship care plans that address these needs including earlier interventions in the form of pre-habilitation as well as survivorship programs is evident (11, 20, 57, 58).

Results observed here, point to the need to develop multidisciplinary survivorship care teams that include mental health care experts who can diagnose and treat patients and survivors in need of mental health support. They also point to the need to develop patient education and empowerment interventions for programs and survivors that address directly their psychosocial oncological needs, including, but not limited to, unmet emotional needs (e.g., loss of sexual function and unmet intimacy needs), social isolation, and lack of social support (11, 20, 49, 59). Results here indicate that preventative efforts to reduce the mental health burden during PCa survivorship are warranted.

## Data Availability Statement

The datasets generated for this article were generated by the Canadian Longitudinal Study of Aging; researchers apply for data access by following the procedure outlined here: www.clsa-elcv.ca. Questions regarding access the datasets should be directed to access@clsa-elcv.ca.

## Ethics Statement

The studies involving human participants were reviewed and approved by Health Sciences Research Ethics Board (#2018-4523), Dalhousie University, Nova Scotia, Canada. The patients/participants provided their written informed consent to participate in this study.

## Author Contributions

GI, LM, RR, SK, and PA: conception, methodology, results, discussion, and reviewed final manuscript. LM, GI, and RR: literature research. GI and LM: statistical analysis and manuscript draft preparation. All authors: contributed to the article and approved the submitted version.

## Conflict of Interest

The authors declare that the research was conducted in the absence of any commercial or financial relationships that could be construed as a potential conflict of interest.

## Abbreviations

PCa: prostate cancer
CLSA: Canadian Longitudinal Study on Aging.

## References

[1] Canadian Cancer Society Canadian Cancer Statistics. (2017). Available online at: http://www.cancer.ca/~/media/cancer.ca/CW/cancer%20information/cancer%20101/Canadian%20cancer%20statistics/Canadian-Cancer-Statistics-2017-EN.pdf (accessed June 15, 2020).

[2] Pernar CH Ebot EM Wilson KM. The epidemiology of prostate cancer. Cold Spring Harb Perspect Med. (2018) 8:a030361. 10.1101/cshperspect.a030361 29311132 PMC6280714

[3] Rawla P. Epidemiology of prostate cancer. World J Oncol. (2019) 10:63–89. 10.14740/wjon1191 31068988 PMC6497009

[4] Nam RK Cheung P Herschorn S Incidence of complications other than urinary incontinence or erectile dysfunction after radical prostatectomy or radiotherapy for prostate cancer: a population-based cohort study. Lancet Oncol. (2014) 15:223–31. 10.1016/S1470-2045(13)70606-5 24440474

[5] Ilie G Rutledge R Sweeney E Anxiety and depression symptoms in adult males in Atlantic Canada with or without a lifetime history of prostate cancer. Psychooncology. (2020) 29:280–6. 10.1002/pon.5244 31652379 PMC7383500

[6] Fervaha G Izard JP Tripp DA Rajan S Leong DP Siemens DR. Depression and prostate cancer: a focused review for the clinician. Urol Oncol. (2019) 37:282–8. 10.1016/j.urolonc.2018.12.020 30630735

[7] Watts S Leydon G Birch B Prescott P Lai L Eardley S Depression and anxiety in prostate cancer: a systematic review and meta-analysis of prevalence rates. BMJ Open. (2014) 13:e003901 10.1136/bmjopen-2013-003901 PMC3963074 24625637

[8] Pasquini M Biondi M. Depression in cancer patients: a critical review. Clin Pract Epidemiol Ment Health. (2007) 3:2. 10.1186/1745-0179-3-2 17288583 PMC1797173

[9] DiMatteo MR Lepper HS Croghan TW Depression is a risk factor for noncompliance with medical treatment: meta-analysis of the effects of anxiety and depression on patient adherence. Arch Intern Med. (2000) 160:2101–7. 10.1001/archinte.160.14.2101 10904452

[10] Prasad SM Eggener SE Lipsitz SR Irwin MR Ganz PA Hu JC. Effect of depression on diagnosis, treatment, and mortality of men with clinically localized prostate cancer. J Clin Oncol. (2014) 32:2471–8. 10.1200/JCO.2013.51.1048 25002728 PMC4121505

[11] Ilie G MacDonald M Bell D An examination of the relationship between mental distress, functional and psychosocial quality of life indicators in a sample of prostate cancer survivors who received curative treatment. Urol Pract. (2019) 7:384–90. 10.1097/UPJ.000000000000000104 37296547

[12] Barnett K Mercer SW Norbury M. Epidemiology of multimorbidity and implications for health care, research, and medical education: a cross-sectional study. Lancet. (2012) 380:37–43. 10.1016/S0140-6736(12)60240-2 22579043

[13] Nieder C Dalhaug A Pawinski A Comorbidity, use of common medications, and risk of early death in patients with localized or locally advanced prostate cancer. ScientificWorldJournal. (2011) 11:1178–86. 10.1100/tsw.2011.121 21666987 PMC5719988

[14] Fortin M Lapointe L Hudon C Vanasse A Ntetu AL Maltais D. Multimorbidity and quality of life in primary care: a systematic review. Health Qual Life Out. (2004) 2:51. 10.1186/1477-7525-2-51 15380021 PMC526383

[15] Read JR Sharpe L Modini M Dear BF. Multimorbidity and depression: a systematic review and meta-analysis. J Affect Disord. (2017) 221:36–46. 10.1016/j.jad.2017.06.009 28628766

[16] Westmaas JL Alcaraz KI Berg CJ Stein KD. Prevalence and correlates of smoking and cessation-related behavior among survivors of ten cancers: findings from a nationwide survey nine years after diagnosis. Cancer Epidemiol Biomarkers Prev. (2014) 23:1783–92. 10.1158/1055-9965.EPI-14-0046 25100826

[17] Zhao J Stockwell T Roemer A. Is alcohol consumption a risk factor for prostate cancer? A systematic review and meta–analysis. BMC Cancer. (2016) 16:845. 10.1186/s12885-016-2891-z 27842506 PMC5109713

[18] Capella MDM Adan A. The age of onset of substance use is related to the coping strategies to deal with treatment in men with substance use disorder. Peer J. (2017) 5:e3660. 10.7717/peerj.3660 28828257 PMC5562142

[19] Spendelow JS Eli Joubert H Lee H Fairhurst BR. Coping and adjustment in men with prostate cancer: a systematic review of qualitative studies. J Cancer Surviv. (2018) 12:155–68. 10.1007/s11764-017-0654-8 29063497 PMC5884891

[20] Ilie G White J Mason R Rendon R Bailly G Lawen J. Current mental distress among men with a history of radical prostatectomy and related adverse correlates. Am J Mens Health. (2020) 14:1557988320957535. 10.1177/1557988320957535 32938266 PMC7503014

[21] Raina P Wolfson C Kirkland S Griffith LE Balion C Cossette B Cohort profile: the Canadian Longitudinal Study on Aging (CLSA). Int J Epidemiol. (2019) 48:1752–3. 10.1093/ije/dyz173 31633757 PMC6929533

[22] Kessler RC Barker PR Colpe LJ Epstein JF Gfroerer JC Hiripi E. Screening for serious mental illness in the general population. Arch Gen Psychiatry. (2003) 60:184–9. 10.1001/archpsyc.60.2.184 12578436

[23] Sampasa-Kanyinga H Zamorski MA Colman I. The psychometric properties of the 10-item Kessler Psychological Distress Scale (K10) in Canadian military personnel. PLoS ONE. (2018) 13:e0196562. 10.1371/journal.pone.0196562 29698459 PMC5919406

[24] Andresen EM Malmgren JA Carter WB Patrick DL. Screening for depression in well older adults: evaluation of a short form of the CES-D (Center for Epidemiologic Studies Depression Scale). Am J Prev Med. (1994) 10:77–84. 8037935

[25] Björgvinsson T Kertz SJ Bigda-Peyton JS McCoy KL Aderka IM. Psychometric properties of the CES-D-10 in a psychiatric sample. Assessment. (2013) 20:429–36. 10.1177/1073191113481998 23513010

[26] Miller WC Anton HA Townson AF. Measurement properties of the CESD scale among individuals with spinal cord injury. Spinal Cord. (2008) 46:287–92. 10.1038/sj.sc.3102127 17909558

[27] Radloff LS CES-D scale: a self report depression scale for research in the general populations. Appl Psychol Measur. (1977) 1:385–401. 10.1177/014662167700100306

[28] Nguyen MT Chan WY Keeler C. The association between self-rated mental health status and total health care expenditure. Medicine. (2015) 94:e1410. 10.1097/MD.0000000000001410 26334899 PMC4616517

[29] Moor I Spallek J Richter M. Explaining socioeconomic inequalities in self-rated health: a systematic review of the relative contribution of material, psychosocial and behavioural factors. J Epidemiol Community Health. (2017) 71:565–75. 10.1136/jech-2016-207589 27682963

[30] Ontario Health Study Available online at: https://www.ontariohealthstudy.ca/ (accessed November 1, 2020).

[31] Tremblay MS Connor Gorber S. Canadian health measures survey: brief overview. Can J Public Health. (2007) 98:453–6. 10.1007/BF03405437 19039881 PMC6976046

[32] Canadian Tobacco Use Monitoring Survey (CTUMS) (2011). Available online at: https://www.canada.ca/en/health-canada/services/publications/healthy-living/canadian-tobacco-use-monitoring-survey-2011-summary.html (accessed November 4, 2020).

[33] Ialomiteanu AR Adlaf EM Hamilton H Mann RE Addiction and Mental Health Indicators Among Ontario Adults 1977-2011. Center for Addiction and Mental Health No.35 (2011). Available online at: https://camh.ca/-/media/files/pdfs—camh-monitor/camh-monitor-2011-trends-ereport-final-pdf.pdf (accessed November 4, 2020).

[34] Sakib MN Shooshtari S St. John P, Menec V. The prevalence of multimorbidity and associations with lifestyle factors among middle-aged Canadians: an analysis of Canadian Longitudinal Study on Aging data. BMC Public Health. (2019) 19:243. 10.1186/s12889-019-6567-x 30819126 PMC6394050

[35] Prados-Torres A Calderón-Larrañaga A Hancco-Saavedra J Poblador-Plou B van den Akker M. Multimorbidity patterns: a systematic review. J Clin Epidemiol. (2014) 67:254–66. 10.1016/j.jclinepi.2013.09.021 24472295

[36] Déruaz-Luyet A N'Goran AA Senn N Bodenmann P Pasquier J Widmer D. Multimorbidity and patterns of chronic conditions in a primary care population in Switzerland: a cross-sectional study. BMJ Open. (2017) 7:e013664. 10.1136/bmjopen-2016-013664 28674127 PMC5734197

[37] World Health Organization Mental Health of Older Adults. (2017). Available online at: https://www.who.int/news-room/fact-sheets/detail/mental-health-of-older-adults (accessed March 23, 2019).

[38] Halpern-Manners A Schnabel L Hernandez EM Silberg JL Eaves LJ The relationship between education and mental health: new evidence from a discordant twin study. Soc Forces. (2016) 95:107–31. 10.1093/sf/sow035

[39] Sareen J Afifi TO McMillan KA Asmundson GJG. Relationship between household income and mental disorders: findings from a population-based longitudinal study. Arch Gen Psychiatry. (2011) 68:419–27. 10.1001/archgenpsychiatry.2011.15 21464366

[40] Spiker RL Mental Health and Marital Status (2014). Available online at: https://www.semanticscholar.org/paper/Mental-Health-and-Marital-Status-Spiker/e34b18f56c1c8b0559c8fb66e3bd17fc0cd5c0dd. 10.1002/9781118410868.WBEHIBS256

[41] Korn EL Graubard BI Analysis of Health Surveys. New York, NY: Wiley (1999).

[42] Canadian Longitudinal Study on Aging (CLSA) CLSA Technical Document. (2017). Available online at: https://www.clsa-elcv.ca/doc/1041 (accessed April 3, 2019).

[43] IBM Method (Multiple Imputation). (2014). Available online at: https://www.ibm.com/support/knowledgecenter/SSLVMB_24.0.0/spss/mva/idh_idd_mi_variables.html (accessed July 28, 2019).

[44] White IR Royston P Wood AM. Multiple imputation using chained equations: Issues and guidance for practice. Stat Med. (2011) 30:377–99. 10.1002/sim.4067 21225900

[45] Enders CK Gottschall AC Multiple imputation strategies for multiple group structural equation models. Struct Equ Model Multidiscip J. (2011) 18:35–54. 10.1080/10705511.2011.532695

[46] Ahmad F Jhajj A K Stewart D E Burghardt M Bierman AS. Single item measures of self-rated mental health: a scoping review. BMC Health Serv Res. (2014) 14:398. 10.1186/1472-6963-14-398 25231576 PMC4177165

[47] Bramness JG Walby FA Hjellvik V Selmer R Tverdal A. Self-reported mental health and its gender differences as a predictor of suicide in the middle-aged. Am J Epidemiol. (2010) 172:160–6. 10.1093/aje/kwq091 20519262

[48] Prostate Cancer Canada TrueNTH. (2019). Available online at: https://app2.connectedwellness.com/start/pcc/index.html?gateway=truenth.ca&nocache=1560294896199 (accessed June 11, 2019).

[49] Ilie G Mason R Bell D Bailley G Rendon RA Mann R Development and initial evaluation of multifaceted intervention to improve mental health and quality of life among prostate cancer survivors. Int J Ment Health AD. (2019) 18:1067–80. 10.1007/s1146

[50] Mental Health Commission of Canada (2014). Overview of Mental Health Data in Canada: Background, Needs, Gaps, Calgary, AB. Copies of this report. Available online at: www.mentalhealthcommission.ca

[51] Nunes BP Flores TR Mielke GI Thumé E Facchini LA. Multimorbidity and mortality in older adults: a systematic review and meta-analysis. Arch Gerontol Geriatr. (2016) 67:130–8. 10.1016/j.archger.2016.07.008 27500661

[52] Müezzinler A Mons U Gellert C Schöttker B Jansen E Kee F. Smoking and all-cause mortality in older adults: results from the CHANCES consortium. Am J Prev Med. (2015) 49:e53–63. 10.1016/j.amepre.2015.04.004 26188685

[53] Sadakane A Gotoh T Ishikawa S Nakamura Y Kayaba K. Amount and frequency of alcohol consumption and all-cause mortality in a Japanese population: the JMS cohort study. J Epidemiol. (2009) 19:107–15. 10.2188/jea.je20081003 19398849 PMC3924134

[54] Johnston MC Crilly C Black C Prescott GJ Mercer SW. Defining and measuring multimorbidity: a systematic review of systematic reviews. Eur J Public Health. (2019) 29:182–9. 10.1093/eurpub/cky098 29878097

[55] Ilie G Rutledge RDH Sweeney E An examination of the role of socioeconomic status in the relationship between depression and prostate cancer survivorship in a population-based sample of men from Atlantic Canada. Oncology. (2020). 10.1159/000512444 33486485

[56] Griffith LE Gilsing A Mangin D Patterson C van den Heuvel E Sohel N. Multimorbidity frameworks impact prevalence and relationships with patient-important outcomes. J Am Geriatr Soc. (2019) 67:1632–40. 10.111/jgs.15921 30957230

[57] Shakeel S Tung J Finley C. Evaluation of factors associated with unmet needs in adult cancer survivors in Canada. JAMA Netw Open. (2020) 3:e200506. 10.1001/jamanetworkopen.2020.0506 32142127 PMC7060489

[58] Nead KT Sinha S Yang DD Nguyen PL. Association of androgen deprivation therapy and depression in the treatment of prostate cancer: a systematic review and meta-analysis. Urol Oncol. (2017) 35:664.e1–4.e9. 10.1016/j.urolonc.2017.07.016 28803700

[59] De Sousa A Sonavane S Mehta J. Psychological aspects of prostate cancer: a clinical review. Prostate Cancer Prostatic Dis. (2012) 15:120–7. 10.1038/pcan.2011.66 22212706

