# Supplementary material for: Assessment of Current Mental Health Status in a Population-Based Sample of Canadian Men With and Without a History of Prostate Cancer Diagnosis: An Analysis of the Canadian Longitudinal Study on Aging (CLSA)
Source: Front Psychiatry. 2020 Dec 16;11:586260. doi: 10.3389/fpsyt.2020.586260 (PMC7772192; doi:10.3389/fpsyt.2020.586260)
Supplement: Supplementary file 1 [file Data_Sheet_1.docx]

Table 1a. Weighted estimates and logistic regression analyses using pooled multiple imputation data examining the association between lifetime history of PCa and psychological distress (K10) for Canadian men from the baseline cycle of the CLSA, 2010-2015.

|  | Multiple Imputation (pooled n = 14 777)^1, MI^ | |
| --- | --- | --- |
|  | Screened positive for psychological distress | |
|  | **No**  (pooled n = 13349) | **Yes**  (pooled n = 1428) |
|  | *F (7, 14757) = 49.65**** | |
| Lifetime history of PCa diagnosis ^MI^ | *F (1, 14763) = 10.08*** | |
| Yes (n) | (728) | (85) |
|  | 3.8 (3.5, 4.1) | 4.7 (3.6, 6.2) |
| aOR^MI^(95% CI) | **1.00 (Reference)** | **1.57 (1.18, 2.08)**** |
| No (n) | (12621) | (1343) |

*** significant at P < 0.001; ** significant at P < 0.01; * significant at P < 0.05 (two-tailed)^;^

^1^Comprehensive cohort only;^a^analyses were controlled for age, province, education, household income, marital status, ethnicity and complexity of the design

^MI^ Multiple imputation, pooled data analysis for 20 iterations which included all variables in the model

Table 2a.Multivariate logistic regression using pooled multiple imputation data predicting current psychological distress (K10) by status of lifetime history of PCa, multimorbidity, alcohol use and smoking for Canadian men from the baseline cycle of the CLSA, 2010-2015.

|  | Screened positive for psychological distress  aORa  95% CI |
| --- | --- |
|  | *F (13, 14751) = 39.87**** |
| Lifetime history of PCa diagnosis | *F (1, 14763) = 10.96*** |
| Yes | 1.62 (1.22, 2.16)** |
| No | 1.00 (Reference) |
| Multimorbidity | *F (1, 14763) = 66.54**** |
| Yes | 1.74(1.52, 1.98)*** |
| No | 1.00 (Reference) |
| Alcohol Use | *F (3, 14761) = 9.98**** |
| Daily drinker | 1.62 (1.32, 1.98)*** |
| Weekly drinker | 1.44 (1.18, 1.75)*** |
| Occasional drinker | 1.12 (0.93, 1.36) |
| Non-drinker | 1.00 (Reference) |
| Smoking | *F (2, 14762) = 31.64**** |
| Daily smoker | 1.85(1.59, 2.15)*** |
| Occasional smoker | 1.28 (0.92, 1.79) |
| Non-smoker | 1.00 (Reference) |

*** significant at P < 0.001; ** significant at P < 0.01; * significant at P < 0.05 (two-tailed)^;^

^1^Comprehensive cohort only;^a^analyses were controlled for age, province, education, household income, marital status, ethnicity and complexity of the design

^MI^ Multiple imputation, pooled data analysis for 20 iterations which included all variables in the model
